# A Case of Strangulated Hernia, in Which an Operation Was Necessary

**Published:** 1851-09

**Authors:** W. J. Owen

**Affiliations:** Port Gibson, Miss.


Art. II.—*A Case of Strangulated Hernia, in which an operation was necessary.* By W. J. Owen, M.D., of Port Gibson, Miss.

In November last, a negro man, slave, aged thirty-three years, stout frame, was found complaining of great pain in the inguinal region, extending to the thigh and abdomen; and attended with considerable nausea. On examination it proved that he had failed to reduce a hernia of which he had been the subject, and that he had been in that condition for forty hours. I was called conjointly with Dr. C. W. Wilson, of this place, and the patient presented the following appearance when we reached him:—A large scrotal tumor in the right side; continual hiccup and retching; pulse tense and full; vomiting, but not stercoraceous.

We resorted to the taxis, warm bath, and cataplasms of tobacco, but without any decided effect, and an operation was decided upon.

When the integuments were laid open, the epiploon escaped, and we found it necessary to excise a portion of this membrane in order to reduce the tumor—a practice condemned by some, I am aware, but attended by no bad results in this case. Eight or ten ounces of serum had been poured out into the peritoneal sac, and lymph was found in patches over the strangulated bowel. The spermatic cord was much enlarged, and on inquiry, I learned from the master that he had been the subject of spermatocele, in consequence of which he was for a good while unable to apply a truss. The operation was successful and the cure complete.

July 1, 1851.

